# Supplementary material for: Elastography Techniques for the Assessment of Liver Fibrosis in Non-Alcoholic Fatty Liver Disease
Source: Int J Mol Sci. 2020 Jun 5;21(11):4039. doi: 10.3390/ijms21114039 (PMC7313067; doi:10.3390/ijms21114039)
Supplement: Supplementary file 1 [file ijms-21-04039-s001.pdf]

International Journal of *Molecular Sciences*

MDPI

Supplementary Table 1. Characteristics of 47 studies included in previously published meta-analyses

Technique	Meta-analyses	Ref. No.	First author	Year	Country	Study design	Patients with NAFLD (n)	Mean age (years)	Mean BMI (m/kg²)	Male sex, n (%)	Histological score	Noninvasive methods
VCTE	Xiao	1	Wong	2008	Hong Kong	Prospective	34	Unclear	Unclear	Unclear	Brunt	M probe
	Kwok, Jiang	2	Yoneda	2008	Japan	Prospective	97	51.8	26.6	40 (41.2%)	Brunt	M probe
	Xiao	3	Friedrich-Rust	2010	Germany	Unclear	50	44	29	27 (54%)	Kleiner	M probe, XL probe
	Kwok, Xiao, Jiang	4	Lupsor	2010	Romania	Prospective	72	42	28.71	51 (70.8%)	Brunt	5-MHz ultrasound transducer probe
	Kwok, Jiang	5	Myers	2010	Canada	Unclear	50	Unclear	Unclear	Unclear	Kleiner	M probe
	Kwok, Xiao, Jiang	6	Wong	2010	France/ Hong Kong	Unclear	246	51	28	135 (54.9%)	Kleiner	M probe, AST/ALT ratio, APRI, BARD score, FIB-4, NFS
	Kwok, Jiang	7	Yoneda	2010	Japan	Unclear	54	50.6	Male, 28.2 Female, 26.2	25 (46.3%)	Brunt	M probe, pSWE, hyaluronic acids, type IV collagen 7S domain
	Kwok, Xiao, Jiang	8	Gaia	2011	Italy	Unclear	72	48	27.5	52 (72.2%)	Brunt	M probe
	Kwok, Xiao	9	Petta	2011	Italy	Unclear	146	44.1	29.1	104 (71%)	Kleiner	M probe
	Xiao, Jiang	10	Myers	2012	Canada	Prospective	127	Unclear	Unclear	Unclear	Kleiner	M probe, XL probe
	Xiao	11	Wong	2012	France /Hong Kong	Unclear	193	Unclear	Unclear	Unclear	Unclear	M probe, XL probe
	Xiao	12	Alkhouri	2013	Italy	Unclear	67 (children)	Unclear	Unclear	46 (68.7%)	Kleiner	S probe
	Xiao	13	Frossard	2013	Switzerland	Prospective	Unclear	Unclear	Unclear	Unclear	Metavir	M probe, AST/ALT ratio, APRI, FIB-4

	Kwok, Xiao, Jiang	14	Kumar	2013	India	Unclear	120	39.1	26.1	90 (75%)	Kleiner	M probe, AST/ALT ratio, APRI, BARD score, FIB-4, NFS
	Xiao	15	Mahadeva	2013	Malaysia	Unclear	131	49.9	Unclear	69 (52.7%)	Kleiner	M probe, APRI
	Xiao	16	Aykut	2014	Turkey	Unclear	88	46	30.3	50 (56.8%)	Kleiner	M probe, XL probe, FibroMeter™ NAFLD, NFS
	Xiao	17	Naveau	2014	France	Prospective	100	42.5	42.3	19 (19%)	Kleiner	M probe, XL probe
	Xiao	18	Chan	2015	Malaysia	Prospective	147	50.5	29.3	80 (54.4%)	Kleiner	M probe, NFS
	Xiao	19	Petta	2015	Italy	Unclear	253	45.2	29.1	177 (70%)	Kleiner	M probe
	Xiao	20	Petta	2015	Italy	Retrospective	321	Unclear	Unclear	Unclear	Kleiner	M probe, AST/ALT ratio, APRI, BARD score, FIB-4, NFS
	Jiang	21	Attia	2016	Germany	Prospective	97	50	31	46 (47.4%)	Kleiner	M probe, XL probe, pSWE
	Xiao	22	Boursier	2016	France	Unclear	452	55.9	31.1	271 (60.0%)	Kleiner	M probe, APRI, BARD score, FIB-4, FibroMeter^{NAFLD}, FibroMeter^{V2G}, FibroTest, NFS, Hepascore
	Jiang	23	Cassinotto	2016	France	Prospective	291	56.7	32.1	172 (59.1%)	Kleiner	M probe, pSWE, 2D-SWE
	Xiao, Jiang	24	Imajo	2016	Japan	Unclear	142	57.5	28.1	81 (57.0%)	Brunt	M probe, MRE
pSWE	Liu, Jiang, Lin	25	Osaki	2010	Japan	Unclear	23	Unclear	Unclear	Unclear	Brunt	pSWE
	Kwok, Jiang	7	Yoneda	2010	Japan	As above.						
	Liu, Jiang, Lin	26	Palmeri	2011	USA	Retrospective/ Prospective	172	Unclear	Unclear	65 (37.8%)	Kleiner	pSWE, APRI
	Liu, Lin	27	Friedrich-Rust	2012	Germany	Unclear	57	45	28	30 (52.6%)	Kleiner	M probe, XL probe, pSWE
	Liu, Lin	28	Guzmán-Aroca	2012	Spain	Prospective	32	43.7	Male, 44.3 Female, 45.1	18 (56.3%)	Brunt, Matteoni	pSWE
	Liu, Jiang	29	Cassinotto	2013	France	Prospective	Unclear	Unclear	Unclear	Unclear	Kleiner	M probe, XL probe, pSWE,

												FibroTest
	Liu, Jiang, Lin	30	Fierbinteanu Braticevici	2013	Romania	Unclear	64	Unclear	Unclear	Unclear	Unclear	pSWE
	Jiang	-	Zhang Da-kun and Yang	2014	Not in PubMed.							
	Lin	31	Guerra	2015	Brazil	Unclear	7	Unclear	Unclear	Unclear	Metavir	pSWE
	Lin	32	Karlas	2015	Germany	Unclear	41	45.7	46	13 (32%)	Kleiner	M probe, XL probe, pSWE, ELF
	Jiang	-	Li	2016	Not in PubMed.							
	Jiang, Lin	21	Attia	2016	Germany	As above.						
	Jiang	23	Cassinotto	2016	France	As above.						
	Lin	33	Cui	2016	USA	Prospective	125	48.9	31.8	57 (45.6%)	Kleiner	pSWE, MRE
	Lin	34	Harris	2016	Australia	Retrospective	Unclear	Unclear	Unclear	Unclear	Metavir	pSWE
	Lin	35	Lee	2017	Korea	Prospective	94	55.5	27.1	41 (43.6%)	Kleiner	M probe, pSWE, 2D-SWE
MRE	Sigh	36	Yin	2007	USA	Unclear	10	Unclear	Unclear	Unclear	Brunt	MRE
	Sigh	37	Asbach	2008	Germany	Unclear	Unclear	Unclear	Unclear	Unclear	Unclear	MRE
	Sigh	38	Asbach	2010	Germany	Prospective	8	Unclear	Unclear	Unclear	Desmet	MRE
	Sigh	39	Chen	2011	USA	Retrospective	58	51.5	38.3	10 (17.2%)	Brunt	MRE
	Sigh	40	Wang	2011	USA	Prospective	8	Unclear	Unclear	Unclear	Brunt	MRE
	Sigh	41	Godfrey	2012	UK	Unclear	7	Unclear	Unclear	Unclear	Kleiner	MRE
	Sigh	42	Rustogi	2012	USA	Retrospective	1	Unclear	Unclear	Unclear	Brunt	MRE
	Xiao	43	Kim	2013	Korea	Retrospective	142	52.8	36.32	38 (26.8%)	Kleiner	MRE, AST/ALT ratio, APRI, BARD score, FIB-4, NFS, Simple panel
	Sigh, Xiao	44	Loomba	2014	USA	Prospective	117	50.1	32.4	51 (43.6%)	Kleiner	MRE
	Xiao	45	Cui	2015	USA	Prospective	102	51.3	31.7	60 (58.8%)	Kleiner	MRE, AST/ALT ratio, APRI, Bonacini CDS, BARD score, FIB-4, Lok index, NASH CRN model, NFS

	Xiao	33	Cui	2016	USA	As above.
	Xiao	24	Imajo	2016	Japan	As above.

NAFLD, non-alcoholic fatty liver disease; BMI, body mass index; AST, aspartate aminotransferase; ALT, alanine aminotransferase; VCTE, vibration-controlled transient elastography; pSWE, point shear wave elastography; 2D-SWE; two-dimensional SWE; MRE, magnetic resonance elastography; APRI, aspartate aminotransferase-to-platelet ratio index; Bonacini CDS, Bonacini cirrhosis discriminant score; ELF, enhanced liver function score; FIB-4, fibrosis-4 index; NASH CRN model, Nonalcoholic Steatohepatitis Clinical Research Network model; NFS, non-alcoholic fatty liver disease fibrosis score.

References

1. Wong, G.L.; Wong, V.W.; Choi, P.C.; Chan, A.W.; Chum, R.H.; Chan, H.K.; Lau, K.K.; Chim, A.M.; Yiu, K.K.; Chan, F.K.; et al. Assessment of fibrosis by transient elastography compared with liver biopsy and morphometry in chronic liver diseases. *Clin Gastroenterol Hepatol* **2008**, *6*, 1027–1035.
2. Yoneda, M.; Yoneda, M.; Mawatari, H.; Fujita, K.; Endo, H.; Iida, H.; Nozaki, Y.; Yonemitsu, K.; Higurashi, T.; Takahashi, H.; et al. Noninvasive assessment of liver fibrosis by measurement of stiffness in patients with nonalcoholic fatty liver disease (NAFLD). *Dig Liver Dis* **2008**, *40*, 371–378.
3. Friedrich-Rust, M.; Hadji-Hosseini, H.; Kriener, S.; Herrmann, E.; Sircar, I.; Kau, A.; Zeuzem, S.; Bojunga, J. Transient elastography with a new probe for obese patients for non-invasive staging of non-alcoholic steatohepatitis. *Eur Radiol* **2010**, *20*, 2390–2396.
4. Lupsor, M.; Badea, R.; Stefanescu, H.; Grigorescu, M.; Serban, A.; Radu, C.; Crişan, D.; Sparchez, Z.; Iancu, S.; Maniu, A. Performance of unidimensional transient elastography in staging non-alcoholic steatohepatitis. *J Gastrointestin Liver Dis* **2010**, *19*, 53–60.
5. Myers, R.P.; Elkashab, M.; Ma, M.; Crotty, P.; Pomier-Layrargues, G. Transient elastography for the noninvasive assessment of liver fibrosis: a multicentre Canadian study. *Can J Gastroenterol* **2010**, *24*, 153986.
6. Wong, V.W.; Vergniol, J.; Wong, G.L.; Foucher, J.; Chan, H.L.; Le Bail, B.; Choi, P.C.; Kowo, M.; Chan, A.W.; Merrouche, W.; et al. Diagnosis of fibrosis and cirrhosis using liver stiffness measurement in nonalcoholic fatty liver disease. *Hepatology* **2010**, *51*, 454–462.
7. Yoneda, M.; Suzuki, K.; Kato, S.; Fujita, K.; Nozaki, Y.; Hosono, K.; Saito, S.; Nakajima, A. Nonalcoholic fatty liver disease: US-based acoustic radiation force impulse elastography. *Radiology* **2010**, *256*, 640–647.
8. Gaia, S.; Carenzi, S.; Barilli, A.L.; Bugianesi, E.; Smedile, A.; Brunello, F.; Marzano, A.; Rizzetto, M. Reliability of transient elastography for the detection of fibrosis in non-alcoholic fatty liver disease and chronic viral hepatitis. *J Hepatol* **2011**, *54*, 64–71.
9. Petta, S.; Di Marco, V.; Cammà, C.; Butera, G.; Cabibi, D.; Craxì, A. Reliability of liver stiffness measurement in non-alcoholic fatty liver disease: the effects of body mass index. *Aliment Pharmacol Ther* **2011**, *33*, 1350–1360.
10. Myers, R.P.; Pomier-Layrargues, G.; Kirsch, R.; Pollett, A.; Duarte-Rojo, A.; Wong, D.; Beaton, M.; Levstik, M.; Crotty, P.; Elkashab, M. Feasibility and diagnostic performance of the FibroScan XL probe for liver stiffness measurement in overweight and obese patients. *Hepatology* **2012**, *55*, 199–208.
11. Wong, V.W.; Vergniol, J.; Wong, G.L.; Foucher, J.; Chan, A.W.; Chermak, F.; Choi, P.C.; Merrouche, W.; Chu, S.H.; Pesque, S.; et al Liver stiffness measurement using XL probe in patients with nonalcoholic fatty liver disease. *Am J Gastroenterol* **2012**, *107*, 1862–1871.

12. Alkhouri, N.; Sedki, E.; Alisi, A.; Lopez, R.; Pinzani, M.; Feldstein, A.E.; Nobili, V. Combined paediatric NAFLD fibrosis index and transient elastography to predict clinically significant fibrosis in children with fatty liver disease. *Liver Int* **2013**, *33*, 79–85.
13. Frossard, J.L.; Giostra, E.; Rubbia-Brandt, L.; Hadengue, A.; Spahr, L. The role of transient elastography in the detection of liver disease in patients with chronic pancreatitis. *Liver Int* **2013**, *33*, 1121–1127.
14. Kumar, R.; Rastogi, A.; Sharma, M.K.; Bhatia, V.; Tyagi, P.; Sharma, P.; Garg, H.; Chandan Kumar, K.N.; Bihari, C.; Sarin, S.K. Liver stiffness measurements in patients with different stages of nonalcoholic fatty liver disease: diagnostic performance and clinicopathological correlation. *Dig Dis Sci* **2013**, *58*, 265–274.
15. Mahadeva, S.; Mahfudz, A.S.; Vijayanathan, A.; Goh, K.L.; Kulenthran, A.; Cheah, P.L. Performance of transient elastography (TE) and factors associated with discordance in non-alcoholic fatty liver disease. *J Dig Dis* **2013**, *14*, 604–610.
16. Aykut, U.E.; Akyuz, U.; Yesil, A.; Eren, F.; Gerin, F.; Ergelen, R.; Celikel, C.A.; Yilmaz, Y. A comparison of FibroMeter™ NAFLD Score, NAFLD fibrosis score, and transient elastography as noninvasive diagnostic tools for hepatic fibrosis in patients with biopsy-proven non-alcoholic fatty liver disease. *Scand J Gastroenterol* **2014**, *49*, 1343–1348.
17. Naveau, S.; Lamouri, K.; Pourcher, G.; Njiké-Nakseu, M.; Ferretti, S.; Courie, R.; Tranchart, H.; Ghinoiu, M.; Balian, A.; Prévot, S.; et al. The diagnostic accuracy of transient elastography for the diagnosis of liver fibrosis in bariatric surgery candidates with suspected NAFLD. *Obes Surg* **2014**, *24*, 1693–1701.
18. Chan, W.K.; Nik Mustapha, N.R.; Mahadeva, S. A novel 2-step approach combining the NAFLD fibrosis score and liver stiffness measurement for predicting advanced fibrosis. *Hepatol Int* **2015**, *9*, 594–602.
19. Petta, S.; Maida, M.; Macaluso, F.S.; Di Marco, V.; Cammà, C.; Cabibi, D.; Craxì, A. The severity of steatosis influences liver stiffness measurement in patients with nonalcoholic fatty liver disease. *Hepatology* **2015**, *62*, 1101–1110.
20. Petta, S.; Vanni, E.; Bugianesi, E.; Di Marco, V.; Cammà, C.; Cabibi, D.; Mezzabotta, L.; Craxì, A. The combination of liver stiffness measurement and NAFLD fibrosis score improves the noninvasive diagnostic accuracy for severe liver fibrosis in patients with nonalcoholic fatty liver disease. *Liver Int* **2015**, *35*, 1566–1573.
21. Attia, D.; Bantel, H.; Lenzen, H.; Manns, M.P.; Gebel, M.J.; Potthoff, A. Liver stiffness measurement using acoustic radiation force impulse elastography in overweight and obese patients. *Aliment Pharmacol Ther* **2016**, *44*, 366–379.
22. Boursier, J.; Vergniol, J.; Guillet, A.; Hiriart, J.B.; Lannes, A.; Le Bail, B.; Michalak, S.; Chermak, F.; Bertrais, S.; Foucher, J.; et al. Diagnostic accuracy and prognostic significance of blood fibrosis tests and liver stiffness measurement by FibroScan in non-alcoholic fatty liver disease. *J Hepatol* **2016**, *65*, 570–578.
23. Cassinotto, C.; Boursier, J.; de Lédinghen, V.; Lebigot, J.; Lapuyade, B.; Cales, P.; Hiriart, J.-B.; Michalak, S.; Bail, B.L.; Cartier, V.; et al. Liver stiffness in nonalcoholic fatty liver disease: a comparison of supersonic shear imaging, FibroScan, and ARFI with liver biopsy. *Hepatology* **2016**, *63*, 1817–1827.
24. Imajo, K.; Kessoku, T.; Honda, Y.; Tomeno, W.; Ogawa, Y.; Mawatari, H.; Fujita, K.; Yoneda, M.; Taguri, M.; Hyogo, H.; et al. Magnetic resonance imaging more accurately classifies steatosis and fibrosis in patients with nonalcoholic fatty liver disease than transient elastography. *Gastroenterology* **2016**, *150*, 626–637.e7.
25. Osaki, A.; Kubota, T.; Suda, T.; Igarashi, M.; Nagasaki, K.; Tsuchiya, A.; Yano, M.; Tamura, Y.; Takamura, M.; Kawai, H.; et al. Shear wave velocity is a useful marker for managing nonalcoholic steatohepatitis. *World J Gastroenterol* **2010**, *16*, 2918–2925.
26. Palmeri, M.L.; Wang, M.H.; Rouze, N.C.; Abdelmalek, M.F.; Guy, C.D.; Moser, B.; Diehl, A.M.; Nightingale, K.R. Noninvasive evaluation of hepatic fibrosis using acoustic radiation force-based shear stiffness in patients with nonalcoholic fatty liver disease. *J Hepatol* **2011**, *55*, 666–672.
27. Friedrich-Rust, M.; Romen, D.; Vermehren, J.; Kriener, S.; Sadet, D.; Herrmann, E.; Zeuzem, S.; Bojunga, J. Acoustic radiation force impulse-imaging and transient elastography for non-invasive assessment of liver fibrosis and steatosis in NAFLD. *Eur J Radiol* **2012**, *81*, e325–e331.
28. Guzmán-Aroca, F.; Frutos-Bernal, M.D.; Bas, A.; Luján-Mompeán, J.A.; Reus, M.; Berná-Serna Jde, D.; Parrilla, P. Detection of non-alcoholic steatohepatitis in patients with morbid obesity before bariatric surgery: preliminary evaluation with acoustic radiation force impulse imaging. *Eur Radiol* **2012**, *22*, 2525–2532.

29. Cassinotto, C.; Lapuyade, B.; Aït-Ali, A.; Vergniol, J.; Gaye, D.; Foucher, J.; Bailacq-Auder, C.; Chermak, F.; Le Bail, B.; de Lédinghen, V. Liver fibrosis: noninvasive assessment with acoustic radiation force impulse elastography—comparison with FibroScan M and XL probes and FibroTest in patients with chronic liver disease. *Radiology* **2013**, *269*, 283–292.
30. Fierbinteanu Braticevici, C.; Sporea, I.; Panaitescu, E.; Tribus, L. Value of acoustic radiation force impulse imaging elastography for non-invasive evaluation of patients with nonalcoholic fatty liver disease. *Ultrasound Med Biol* **2013**, *39*, 1942–1950.
31. Guerra, J.A.; Trippia, M.; Pissaia, A.; Teixeira, B.C.; Ivantes, C.A. Acoustic radiation force impulse is equivalent to liver biopsy to evaluate liver fibrosis in patients with chronic hepatitis C and nonalcoholic fatty liver disease. *Arq Gastroenterol* **2015**, *52*, 234–238.
32. Karlas, T.; Dietrich, A.; Peter, V.; Wittekind, C.; Lichtinghagen, R.; Garnov, N.; Linder, N.; Schaudinn, A.; Busse, H.; Prettin, C.; et al. Evaluation of transient elastography, acoustic radiation force impulse imaging (ARFI), and enhanced liver function (ELF) score for detection of fibrosis in morbidly obese patients. *PloS One* **2015**, *10*, e0141649.
33. Cui, J.; Heba, E.; Hernandez, C.; Haufe, W.; Hooker, J.; Andre, M.P.; Valasek, M.A.; Aryafar, H.; Sirlin, C.B.; Loomba, R. Magnetic resonance elastography is superior to acoustic radiation force impulse for the diagnosis of fibrosis in patients with biopsy-proven nonalcoholic fatty liver disease: a prospective study. *Hepatology* **2016**, *63*, 453–461.
34. Harris, N.; Nadebaum, D.; Christie, M.; Gorelik, A.; Nicoll, A.; Sood, S.; Gibson, R. Acoustic radiation force impulse accuracy and the impact of hepatic steatosis on liver fibrosis staging. *J Med Imaging Radiat Oncol* **2016**, *60*, 587–592.
35. Lee, M.S.; Bae, J.M.; Joo, S.K.; Woo, H.; Lee, D.H.; Jung, Y.J.; Kim, B.G.; Lee, K.L.; Kim, W. Prospective comparison among transient elastography, supersonic shear imaging, and ARFI imaging for predicting fibrosis in nonalcoholic fatty liver disease. *PloS One* **2017**, *12*, e0188321.
36. Yin, M.; Talwalkar, J.A.; Glaser, K.J.; Manduca, A.; Grimm, R.C.; Rossman, P.J.; Fidler, J.L.; Ehman, R.L. Assessment of hepatic fibrosis with magnetic resonance elastography. *Clin Gastroenterol Hepatol* **2007**, *5*, 1207–1213.e2.
37. Asbach, P.; Klatt, D.; Hamhaber, U.; Braun, J.; Somasundaram, R.; Hamm, B.; Sack, I. Assessment of liver viscoelasticity using multifrequency MR elastography. *Magn Reson Med* **2008**, *60*, 373–379.
38. Asbach, P.; Klatt, D.; Schlosser, B.; Biermer, M.; Muche, M.; Rieger, A.; Loddenkemper, C.; Somasundaram, R.; Berg, T.; Hamm, B.; et al. Viscoelasticity-based staging of hepatic fibrosis with multifrequency MR elastography. *Radiology* **2010**, *257*, 80–86.
39. Chen, J.; Talwalkar, J.A.; Yin, M.; Glaser, K.J.; Sanderson, S.O.; Ehman, R.L. Early detection of nonalcoholic steatohepatitis in patients with nonalcoholic fatty liver disease by using MR elastography. *Radiology* **2011**, *259*, 749–756.
40. Wang, Y.; Ganger, D.R.; Levitsky, J.; Sternick, L.A.; McCarthy, R.J.; Chen, Z.E.; Fasanati, C.W.; Bolster, B.; Shah, S.; Zuehlsdorff, S.; et al. Assessment of chronic hepatitis and fibrosis: comparison of MR elastography and diffusion-weighted imaging. *AJR Am J Roentgenol* **2011**, *196*, 553–561.
41. Godfrey, E.M.; Patterson, A.J.; Priest, A.N.; Davies, S.E.; Joubert, I.; Krishnan, A.S.; Griffin, N.; Shaw, A.S.; Alexander, G.J.; Allison, M.E.; et al. A comparison of MR elastography and 31P MR spectroscopy with histological staging of liver fibrosis. *Eur Radiol* **2012**, *22*, 2790–2797.
42. Rustogi, R.; Horowitz, J.; Harmath, C.; Wang, Y.; Chalian, H.; Ganger, D.R.; Chen, Z.E.; Bolster, B.D. Jr.; Shah, S.; Miller, F.H. Accuracy of MR elastography and anatomic MR imaging features in the diagnosis of severe hepatic fibrosis and cirrhosis. *J Magn Reson Imaging* **2012**, *35*, 1356–1364.
43. Kim, D.; Kim, W.R.; Talwalkar, J.A.; Kim, H.J.; Ehman, R.L. Advanced fibrosis in nonalcoholic fatty liver disease: noninvasive assessment with MR elastography. *Radiology* **2013**, *268*, 411–419.
44. Loomba, R.; Wolfson, T.; Ang, B.; Hooker, J.; Behling, C.; Peterson, M.; Valasek, M.; Lin, G.; Brenner, D.; Gamst, A.; et al. Magnetic resonance elastography predicts advanced fibrosis in patients with nonalcoholic fatty liver disease: a prospective study. *Hepatology* **2014**, *60*, 1920–1928.

45. Cui, J.; Ang, B.; Haufe, W.; Hernandez, C.; Verna, E.C.; Sirlin, C.B.; Loomba, R. Comparative diagnostic accuracy of magnetic resonance elastography vs. eight clinical prediction rules for non-invasive diagnosis of advanced fibrosis in biopsy-proven non-alcoholic fatty liver disease: a prospective study. *Aliment Pharmacol Ther* **2015**, *41*, 1271–1280.
